# Global trends in research related to emergence delirium, 2012–2021: A bibliometric analysis

**DOI:** 10.3389/fpsyg.2023.1098020

**Published:** 2023-03-08

**Authors:** Kenru Wang, Jiehui Cai, Ruiming Du, Jiaxuan Wu

**Affiliations:** ^1^Department of Anesthesia, The Second Affiliated Hospital of Shantou University Medical College, Shantou, Guangdong, China; ^2^Department of General Surgery, The Second Affiliated Hospital of Shantou University Medical College, Shantou, Guangdong, China

**Keywords:** emergence delirium, VOSviewer, CiteSpace, bibliometrix, global trends

## Abstract

**Introduction:**

Emergence delirium is a kind of mental disorder during the early awakening period after general anesthesia, which is manifested as the combination of perceptual disturbance and psychomotor agitation. It is an independent risk factor for postoperative delirium and even long-term postoperative cognitive decline, which often affects the postoperative outcome and deserves the attention of clinical anesthesiologists. There are many studies on emergence delirium, but the quantity and quality of these studies are unclear. Therefore, we conducted a bibliometric analysis of studies on emergence delirium between January 2012 and December 2021. Through the analysis of relevant literature, the research hotspots and trends of emergence delirium are understood, which can provide a reference for future research.

**Methods:**

We searched the Web of Science Core Collection (WoSCC) for original articles and reviews related to emergence delirium published between 2012 and 2021, and collected a variety of bibliographic elements, including annual publications, authors, countries/regions, institutions, journals, and keywords. Three different science-based tools (CiteSpace, VOSviewer and Bibliometrix) were used for this comprehensive analysis.

**Results:**

From January 2012 to December 2021, a total of 912 emergence delirium (ED) related literature were published, including 766 original research articles and 146 review articles. The number of publications has increased every year except 2016. The United States published 203 articles, ranking first with China, followed by South Korea (95 articles). The United States is also the country with the most citations (4,508), and Yonsei Univ is the most productive institution. The most published journal was PEDIATRIC ANESTHESIA, with the highest h and g index. LEE JH is the most influential author in this field.

**Discussion:**

“Children, emergence agitation, delirium, dexmedetomidine” are the hot topics in this field in recent years. The bibliometric analysis in this field will provide the future direction for the study of emergence delirium for clinicians.

## 1. Introduction

Delirium is an acute fluctuating change in mental state, marked by decreased levels of consciousness and disturbances in attention (Oh et al., 2017; Keenan and Jain, 2022). The very early delirium that occurs immediately after anesthesia is called emergence delirium. Emergence delirium (ED) is a confused, excited and fidgety state, which is a potentially dangerous clinical phenomenon. The 2017 Guidelines of the European Society of Anesthesiology (ESA) (Aldecoa et al., 2017) specifically define postoperative delirium from immediately after the end of anesthesia to 24 h after the end of anesthesia as emergence delirium. Emergence delirium often leads to incision site damage, patient and personnel injury, and brings poor prognosis for patients, which can be divided into short-term poor prognosis and long-term poor prognosis. In the short term, it may lead to a prolonged hospital stay (Xará et al., 2013; Choi et al., 2017), increased medical costs, reduced quality of life, etc. Long-term adverse prognosis includes postoperative delirium and long-term cognitive dysfunction (POCD) (Neufeld et al., 2013), increased post-discharge mortality (Veiga et al., 2012) and other serious consequences.

Studies have reported a wide variation in the incidence of emergence delirium in adults, with an observational study involving 91 patients reporting a 45% incidence using DSM-IV diagnosis and a study involving 1,000 patients using Nu-DESC reporting a 4.3% incidence. The large differences in reported incidence among different studies may be related to the large differences in sample size, different diagnostic tools and control of risk factors. Many risk factors are associated with emergence delirium, such as surgical procedure, the score of PAB (pediatric anesthesia behavior), time of anesthesia, age, etc. (Hino et al., 2017). Age is an important factor affecting the occurrence of delirium, and the incidence of delirium is high in children and elderly patients. Franck et al. (2016) found in their study that the average age of people with emergence delirium was higher than those without emergence delirium, indicating that it may be related to the degeneration of brain function in the aged. Some scholars reported that low activity delirium was significantly higher than high activity delirium and mixed delirium in elderly patients with emergence delirium (Card et al., 2015). The incidence of emergence delirium in children is as high as 10–80% (Chandler et al., 2013). Compared with adults, preschool children are more common and widespread (Smessaert et al., 1960). Scholars at home and abroad have conducted a lot of research on it, but the specific mechanism is still unclear.

Bibliometric analysis is a quantitative and qualitative analysis method. It uses mathematical and statistical tools to evaluate the correlation between publications in a certain field, and can objectively analyze the research impact, so as to evaluate its development trend and summarize the research direction (Cancino et al., 2017; Chen, 2017). Although studies have been completed or are ongoing, emergence delirium seems to be underappreciated in clinical practice. At present, there are no scientific reports on bibliometric analyses of high-quality and highly cited papers on emergence delirium. Therefore, through bibliometric analysis of relevant articles in this field, we analyzed the development, trends and research hotspots of emergence delirium in order to clarify the current research status. The purpose of our analysis is to obtain the annual publications and citations, the most productive countries and authors, influential journals, hot topics and keyword analysis information, hoping that the research results can provide useful information to clinical anesthesiologists and relevant researchers in this field, and thus arouse their attention to this field. To provide help for the follow-up mechanism and treatment research.

## 2. Materials and methods

### 2.1. Data source and collection

**Figure 1:**
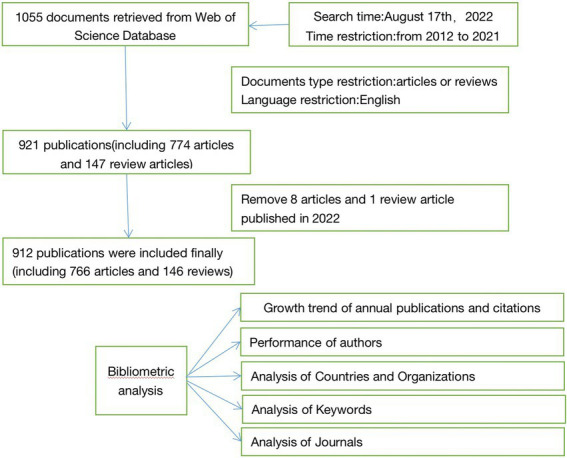
Flowchart of data collection and analysis.

Web of Science, as a high-quality digital literature resource database, is considered the most suitable database for bibliometric analysis due to its powerful functions (He et al., 2022). We searched all publications related to emergence delirium from the Scientific Network Core collection database of Clarivate analysis, including versions of SCI-EXPANDED (2003-present), SSCI (2003-present), AHCI (2003-present), ESCI (2017-present), CCR-Expanded (1985-present), and IC (1993-present). We use the medical subject heading (Mesh) and the entry term “emergence delirium” as the search strategy. Search queries include the following: TS = (Delirium, Emergence) OR TS = (“Anesthesia Emergence Delirium”) OR TS = (Delirium, Anesthesia Emergence) OR TS = (Emergence Delirium, Anesthesia) OR TS = (“Emergence Excitement”) OR TS = (Excitement, Emergence) OR TS = (“Postanesthetic Excitement”) OR TS = (Excitement, Postanesthetic) OR TS = (“Agitated Emergence”) OR TS = (Emergence, Agitated) OR TS = (“Emergence Agitation”), agitated) OR TS = (“Emergence Agitation”), and then screen the time span of these publications from 2012 to 2021. The study was conducted on September 17, 2022 and produced a total of 1,055 documents. We then set the document type to article or review, limited the language to English, and got 921 publications, including 774 articles and 147 review articles. A total of 134 publications were excluded: 45 non-English articles, seven non-English review articles, 29 conference abstracts, 18 editorial materials, 29 letters, four revisions, and two withdrawn publications. Using Zotero software to manage the literature collection, we found nine published articles in 2022 and then excluded them. Finally, the number of publications included in the bibliometric analysis was 912, including 766 articles, accounting for 83.99% of the total, and 146 review articles (16.01%). The detailed filter program is shown in Figure 1.

### 2.2. Data collection

Raw data is downloaded *via* WoSCC. All records retrieved were downloaded in “plain text” format and relevant information was extracted: number of papers, title, keywords, Hirsch index (H-index), country/region and citations, branches, authors, journals and references, etc. Three different sciometrics tools (CiteSpace,V.6.1.R3, VOSviewer1.6.18, and Bibliometrix) were used for knowledge mapping.

### 2.3. Data analysis

The online analysis and retrieval functions from Web of Science(Wos) include: research field, document type, journal, author, affiliate, country, language and the number of articles cited, citation times, etc. The information we gleaned from these publications, including titles, authors, affiliations, languages, document types, abstracts, keywords and citation references, is imported into Biblioshiny (a web-based interface for bibliometric measurement), the VOSviewer software and the CiteSpace software, to get visual analysis and knowledge mapping.

Bibliometrix is a comprehensive R language package for bibliometric analysis and scientific visualization, which is developed and maintained by Massimo Aria and Corrado Cuccurullo. Bibliometrix is used for computation and visualization to extract bibliographic information for descriptive analysis (Aria and Cuccurullo, 2017). The data retrieved from WoS was imported into the Biblioshiny website to obtain key information about these publications and generate images to visualize the results (Aria and Cuccurullo, 2017) including time span, number of sources, type of literature, content of literature, number of literature, number of references, and author. In addition, annual scientific output, average number of article citations per year, sources (most relevant sources, journal sources, and source dynamics), most cited literature and references, Authors (most relevant authors, Authors’ Production over Time, author impact by H-index, G-index, and M-index), contributions by states and affiliates, and keywords are also included. The three stages of bibliometric analysis process were supported: first, data import and conversion to R format; second, bibliometric analysis of data sets; third, construction of matrix. Bibliometrix requires the user to have knowledge of programming in R language and requires a customized interface to be programmed, if desired (Arruda et al., 2022).

VOSviewer is a software tool for constructing and visualizing document metering networks. This software can extract country, keyword, author, and other relevant information from literature retrieval data, and use it for relationship construction and visual analysis, realize the drawing of a scientific knowledge map (Bertoli-Barsotti and Lando, 2017; Yu et al., 2020), and display the co-occurrence analysis of literature keywords, subject words, author and other information. And provides text mining capabilities (Van Eck and Waltman, 2010). VOSviewer explores co-occurrence, citation, co-authorship, bibliographic coupling, and co-cited links in one of three possible representations: network, density, or overlay visualization (Arruda et al., 2022).

CiteSpace is a data standardization method based on set theory, which is used to measure the similarity of knowledge units and obtain time zone and timeline views in time slices to understand the development process and trend of this field. It focuses on identifying key points in the development of a field or domain, especially knowledge turning points and key points. It helps to get the reference pulse of the reference and find keywords with strong reference pulse (Chen, 2006).We exported the retrieved articles in plain text format with complete records and citations under the name “download_XXX.txt” and then imported into CiteSpace,V.6.1.R3 for further analysis (Zhong et al., 2022). Central concepts of CiteSpace includes burst detection, betweenness centrality, and heterogeneous networks, which helps to visualize the current status, hotspots, and frontiers of research in a timely manner.

## 3. Results

### 3.1. Annual publication output growth trend and citation analysis

**Figure 2:**
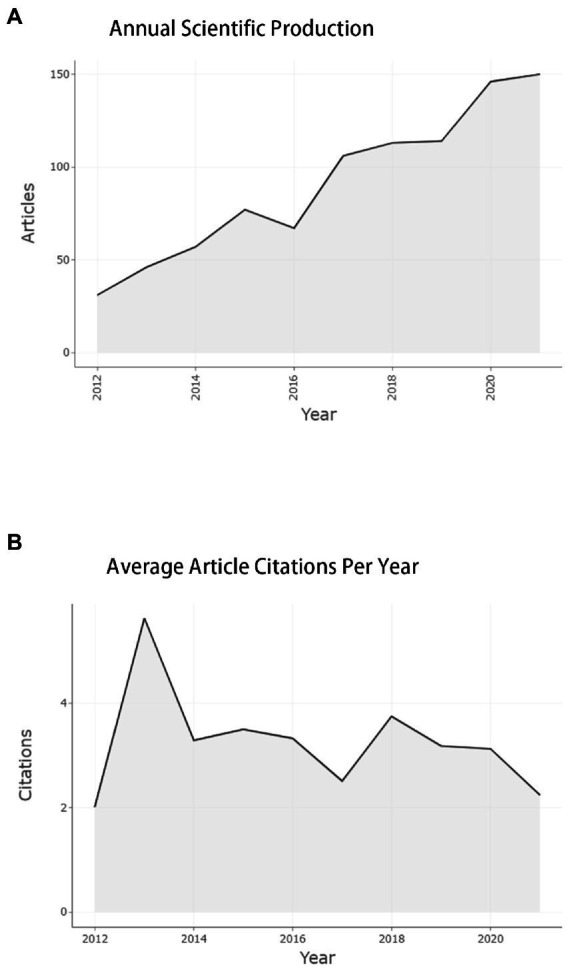
Annual scientific production and average article citations per year from 2012 to 2021 in this research field. **(A)** Annual scientific production from 2012 to 2021 in this research field. **(B)** Average article citations per year from 2012 to 2021 in this research field.

**Figure 3:**
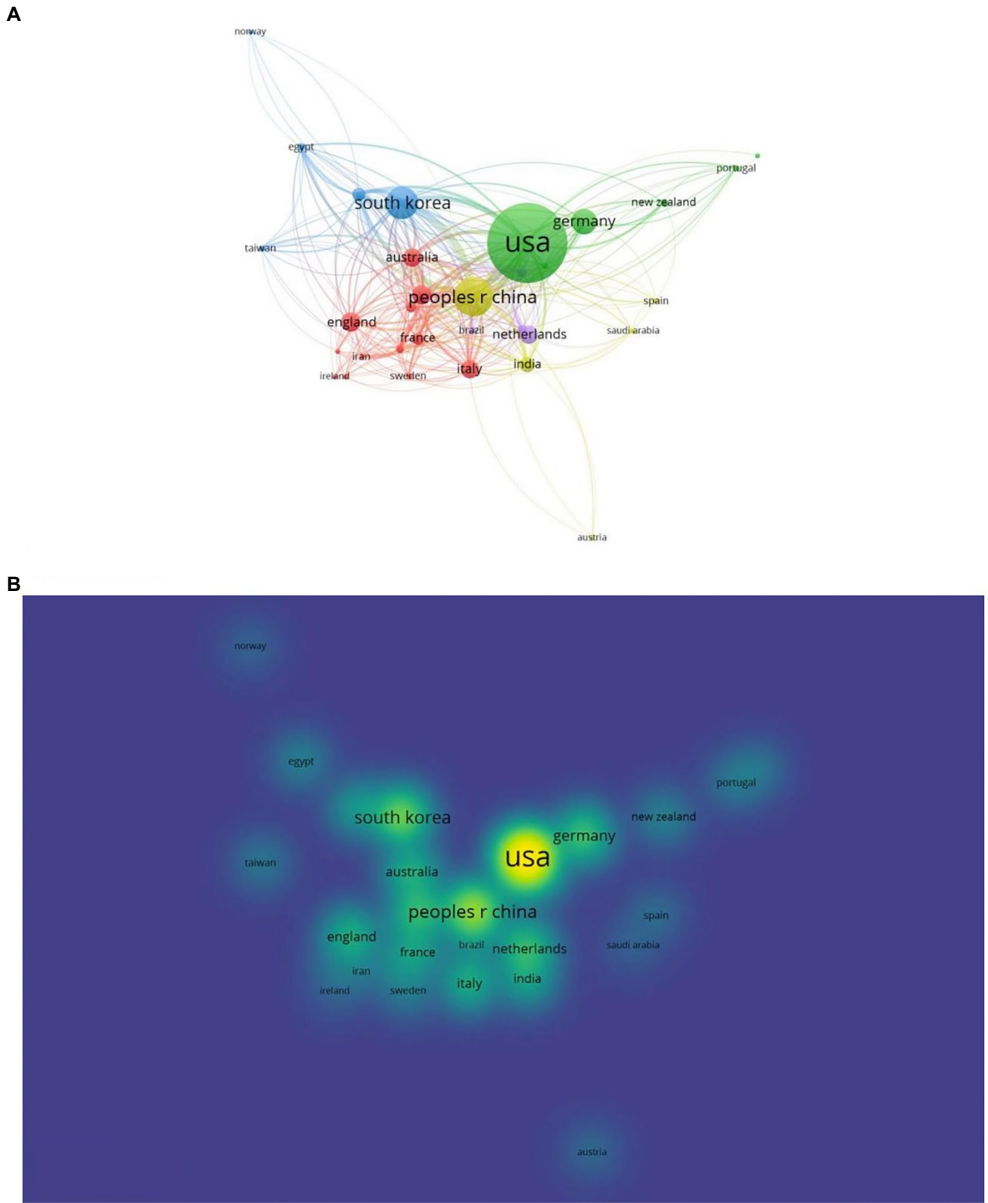
Mapping on countries of studies related to emergence delirium. **(A)** Network map of citation between countries with more than 5 citations. **(B)** Density map of citation between countries with more than 5 citations.

In the past 10 years, the total number of documents related to this topic retrieved from WoS is 912. The overall trend of publication output is increasing year by year, with an annual growth rate of 19.15%, as shown in Figure 2A. Except that 2016 is less than 2015, 2021 will be the year with the largest publication output (150, 16.54%). The number of documents issued rose from 31 (3.40%) in 2012 to 150 (16.54%) in 2021. Citation analysis is to reveal the quantitative characteristics and internal laws of the country, treatise, paper and research institution by analyzing them. The total number of citations is 26,585 (no self-citations are 26,547). The number of citations of an article reflects its scientific influence (Sevinc, 2004). Figure 2B shows the average number of article citations from 2012 to 2021, with the most citations in 2013 (5.62) and the least in 2012 (2.02). Figure 3 represents the national network diagram conducted by VOSviewer for these document citations. Using 5 as the minimum number of citations of a country, 32 countries met the threshold. The more articles published in a country are cited, the larger the node (Figure 3A). A yellower color means more citations (Figure 3B). There are five clusters of the citations in Figure 3A. The top cluster with 12 items suggests the most attractive research field, which is shown in red. In Figure 3, articles written in the United States are cited the most, 4,508 times, followed by China (1,714 citations) and South Korea (1,279 citations). In addition, it is found that the research cooperation with China is the highest in citation analysis.

### 3.2. Performance of authors

In our data set, a total of 3,743 authors participated in this study, including 58 authors of single-authored documents and 3,685 authors of multiauthored documents. Table 1 shows the top 10 prolific authors, LEE JH was the most productive author and he contributed to 14 publications, accounting for 1.54% of all, followed by KIM HS with 13 publications (1.43%) and then ZHANG Y (*n* = 10, 1.10%) and KIM JT (*n* = 8, 0.88%). Based on the h-index, LEE JH was the top 1 (h-index =7), KIM HS and ZHANG Y ranked No. 2 (*h*-index = 6). M-index is used to eliminate the influence of different experiences or ages among authors, helping to identify truly successful scientists (Aria and Cuccurullo, 2017). GARCIA PS has the highest *m*-index of 0.833 and then ZHANG Y (*m*-index = 0.75), LEE JH (*m*-index = 0.7), LI J (*m*-index = 0.556), and KIM HS (*m*-index = 0.545) followed him.

**Table 1:** Top 10 contributing authors in the field of emergence delirium.

Authors	Articles	Articles fractionalized	h_index	g_index	m_index	TC	NP	PY_start
Lee JH	14	2.36	7	14	0.7	225	14	2013
Kim HS	13	2.02	6	8	0.545	89	12	2012
Zhang Y	10	1.54	6	9	0.75	100	9	2015
Dahmani S	5	1.05	5	5	0.455	194	5	2012
Garcia PS	6	1.08	5	6	0.833	120	6	2017
Ingelmo PM	5	1.01	5	5	0.5	155	5	2013
Kim H	7	1.28	5	5	0.5	168	5	2013
Kim JT	8	1.36	5	8	0.455	64	8	2012
Kim SY	7	1.30	5	7	0.455	250	7	2012
Li J	7	1.35	5	7	0.556	92	7	2014

**Figure 4:**
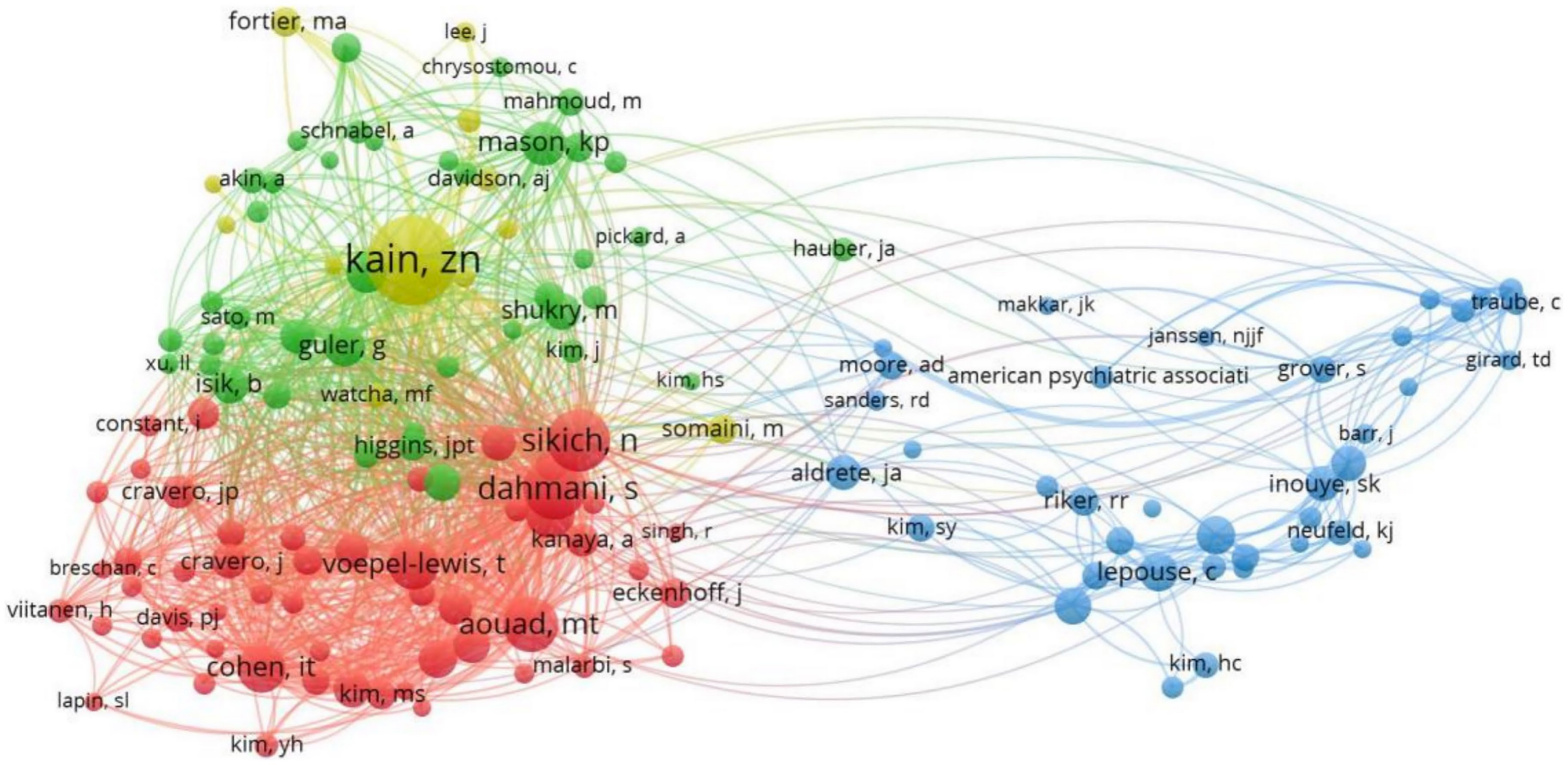
Network map of co-citation between authors with more than 20 citations.

We use VOSviewer software to perform co-citation analysis of cited authors (Figure 4). One hundred forty five authors were analyzed with a minimum of citations of over 20 times. The top 5 authors with the highest link strength in co-citation analysis were Kain’s (total link strength = 5,216), Dahmani’s (total link strength =3,248), Sikichn (total link strength = 2,826), Aouadmt (total link strength =2,746), and Cohen, it (total link strength = 2,503).

### 3.3. Analysis of countries and organizations

**Figure 5:**
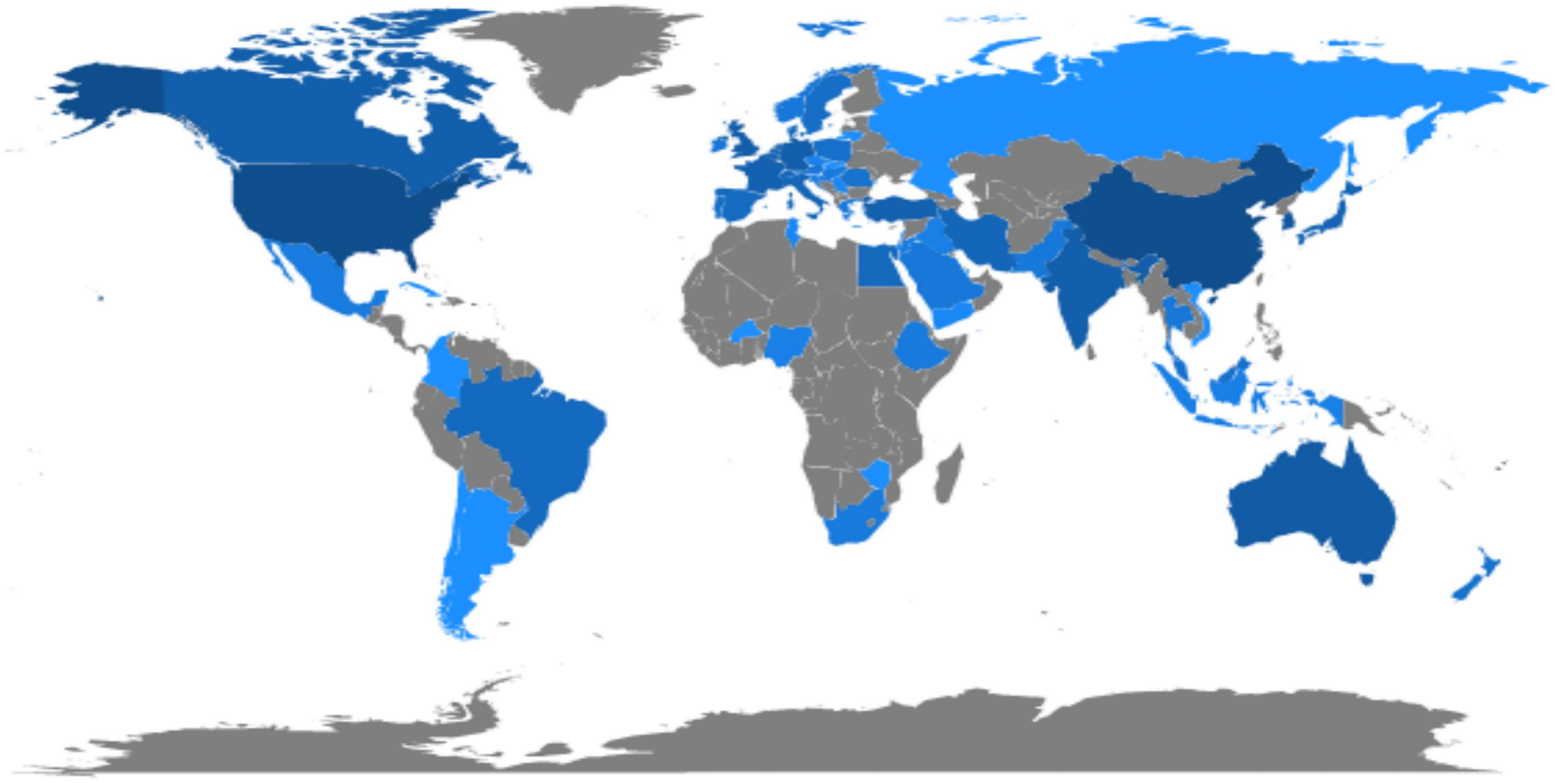
The regional distribution.

**Figure 6:**
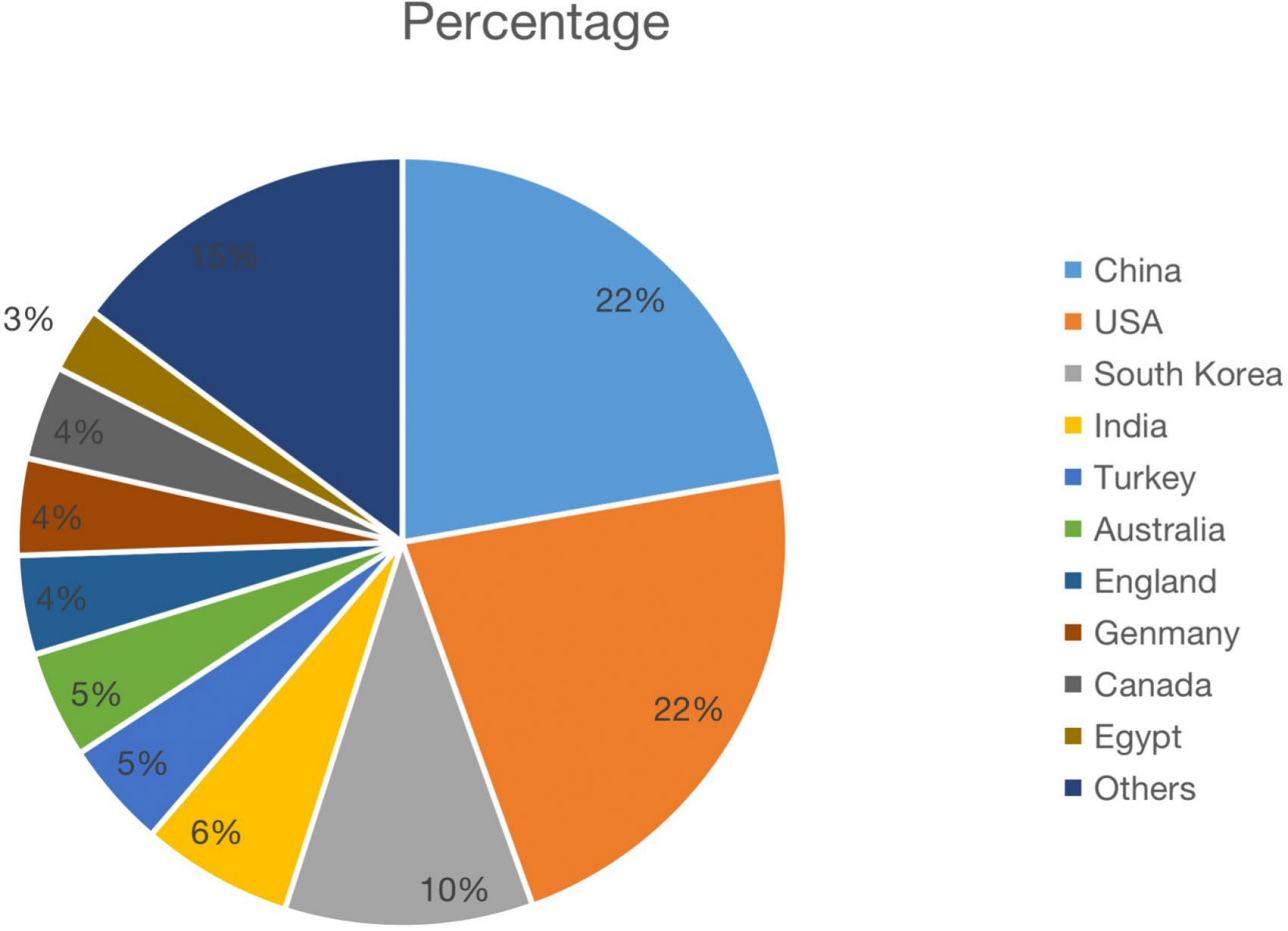
Distributions and percentages of countries/regions of publications.

The 912 literature retrieved in WOS were widely distributed, up to 67 countries, and the average citation times (including self-citation) were 14.29. Global contribution of publications on emergence delirium as shown in Figure 5, the United States, China and South Korea have darker color blocks than other countries and regions, indicating greater contribution to research on emergence delirium. Figure 6 shows that the top 10 countries for all publications from 2012 to 2021 are the United States (203 articles), China (203 articles), South Korea (95 articles), India (58 articles), Turkey (41 articles), Australia (41 articles), England (38 articles), Germany (37 articles), Canada (36 articles), and Egypt (25 articles), a total of 777 articles. According to the increasing trends in different countries, the United States is leading the way in the study of emergence delirium, making the largest contribution and showing a steady upward trend. A total of 1,194 institutions appeared in the selected literature, as shown in Table 2. Among the institutions, Yonsei Univ in Korea published 41 articles and was the most productive institution in the study of emergence delirium between 2012 and 2021.

**Table 2:** Top 10 most productive organizations in this research field.

Rank	Institution	Country	No. of articles
1	Yonsei Univ	Korea	41
2	Sichuan Univ	China	31
3	Seoul Natl Univ	Korea	27
4	Harvard Med Sch	United States	23
5	Korea Univ	Korea	19
6	Univ Michigan	United States	18
7	Monash Univ	Australia	17
8	Wenzhou Med Univ	China	17
9	Katholieke Univ Leuven	Belgium	16
10	Shang hai Jiao Tong Univ	China	16

### 3.4. Analysis of keywords

**Figure 7:**
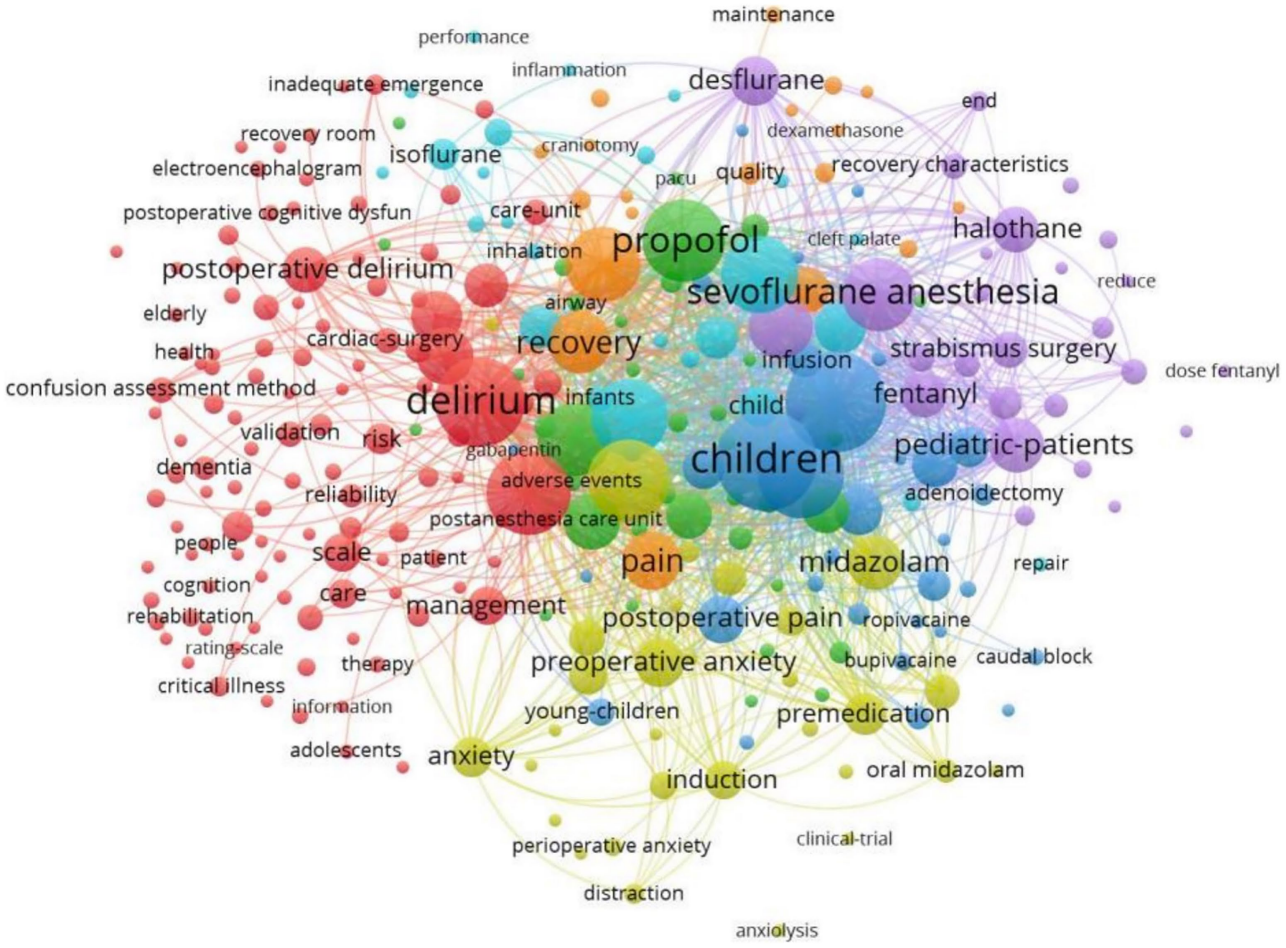
Network map of co-occurrence between keywords with more than five times.

Keywords embody the core and essence of a paper, and are also the concentration of a scientific research field, Keywords plus is a word or phrase that often appears in the title of the reference cited by the retrieved article (Aria et al., 2021). VOSviewer was used to draw the network view of keyword co-occurrence for 912 documents, and 281 important keywords with frequency more than or equal to 5 were selected for visualization. Keywords with higher correlations are more likely to be grouped into the same category with the same color. The results are shown in Figure 7. the identified keywords were divided into 7 clusters. Keywords clustered in the purple area mainly described topics related to sevoflurane anesthesia. In the green area, clustered keywords were related to propofol; In the dark blue area, clustered keywords were related to children; In the light blue area, clustered keywords were related to surgery; In the orange area, clustered keywords were related to children or emergence agitation In general-anesthesia. In yellow and red area, keywords were related to anesthesia and delirium, respectively.

**Figure 8:**
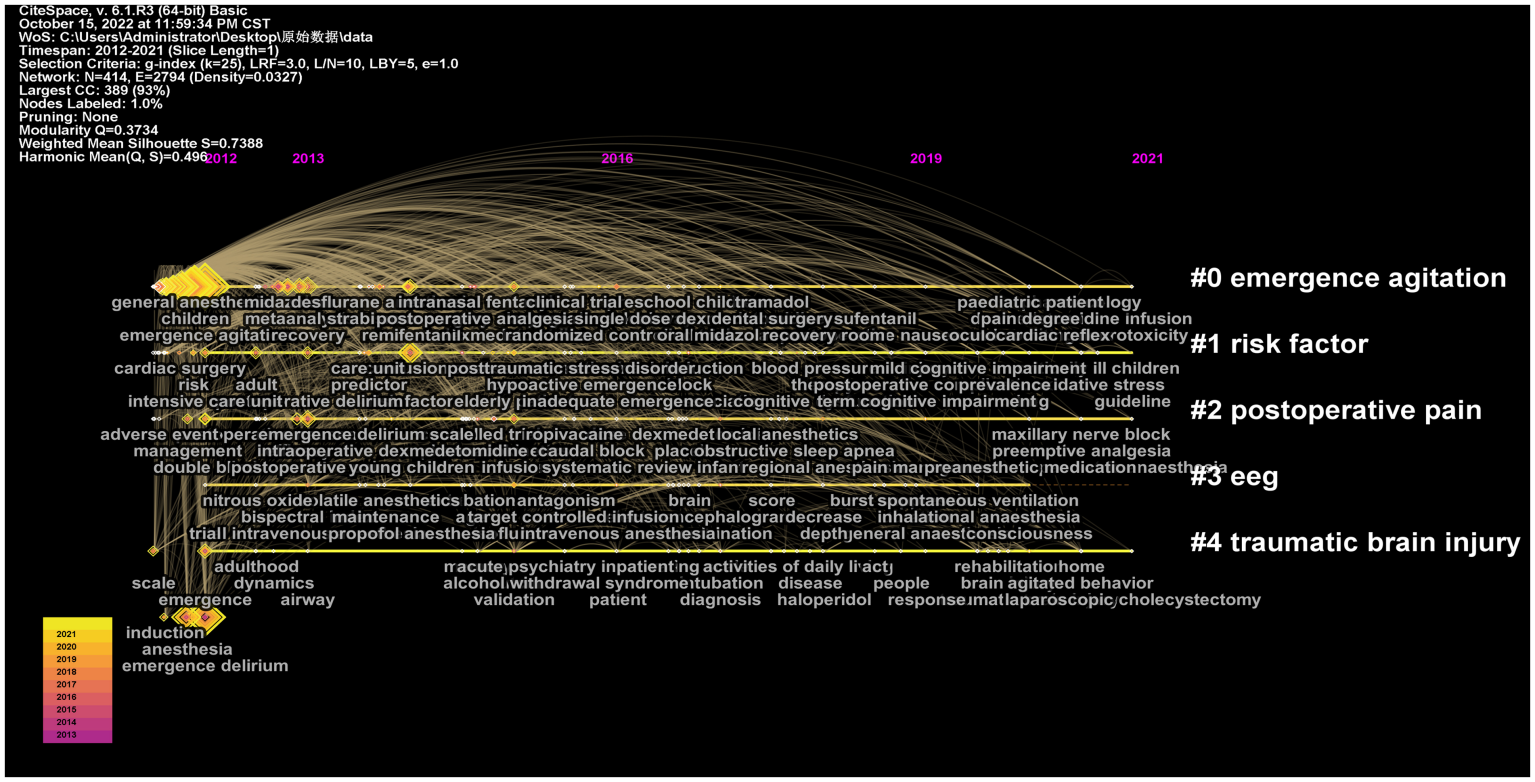
Mapping of the timezone view of recent 10 years on emergence delirium according to CiteSpace.

In order to understand the progress of emergence delirium research in the time dimension the Timezone graph provided by CiteSpace was used to present the map of the keyword co-occurrence network (Figure 8). The size of the cluster label is proportional to the size of the cluster. Each cluster represents a collection of different specialties or topics (von Bohlen Und Halbach, 2011). The graph is divided into five clusters Cluster 0 (study of emergence agitation) Cluster 1 (study of risk factor) Cluster 2 (study of pain) cluster 3 (study of EEG) and cluster 4 (traumatic brain Study of injury). Cluster 0 and cluster 4 are relatively early research directions

**Figure 9:**
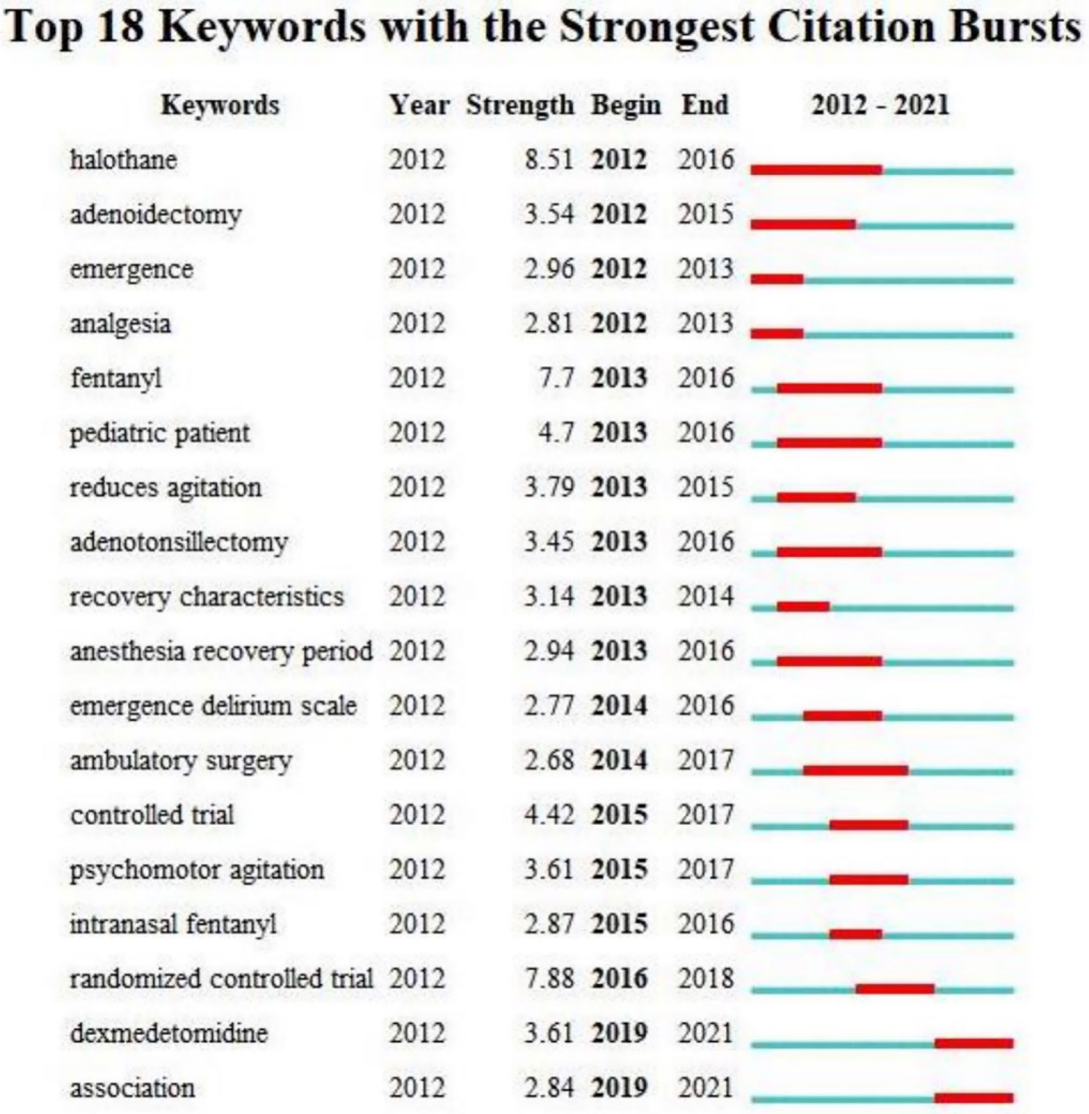
Top 18 keywords with strong citation burstness. The red bars mean some keywords are cited frequently; the blue bars represent keywords cited infrequently.

In this study, CiteSpace was also used to evaluate the first 18 mutant words and their citation history (Figure 9). The so-called emergent word refers to the word that is frequently cited in a period of time, which is an indicator of research frontier topics (Aria and Cuccurullo, 2017). The first three mutant words are halothane,2012, with a mutation value of 8.51; randomized controlled trial,2012, the mutation value was 7.88; fentanyl, 2012, the mutation value is 7.7. It also shows the duration of these emergent objects to see their impact on the status quo. Prior to 2016, research on emergence delirium focused on anesthesia-related risk factors (e.g., narcotic drugs, type of surgery, analgesia, pediatric patients, etc.). However, since 2016, the focus has been on the prevention and mitigation of emergence delirium.

### 3.5. Journal analysis

**Figure 10:**
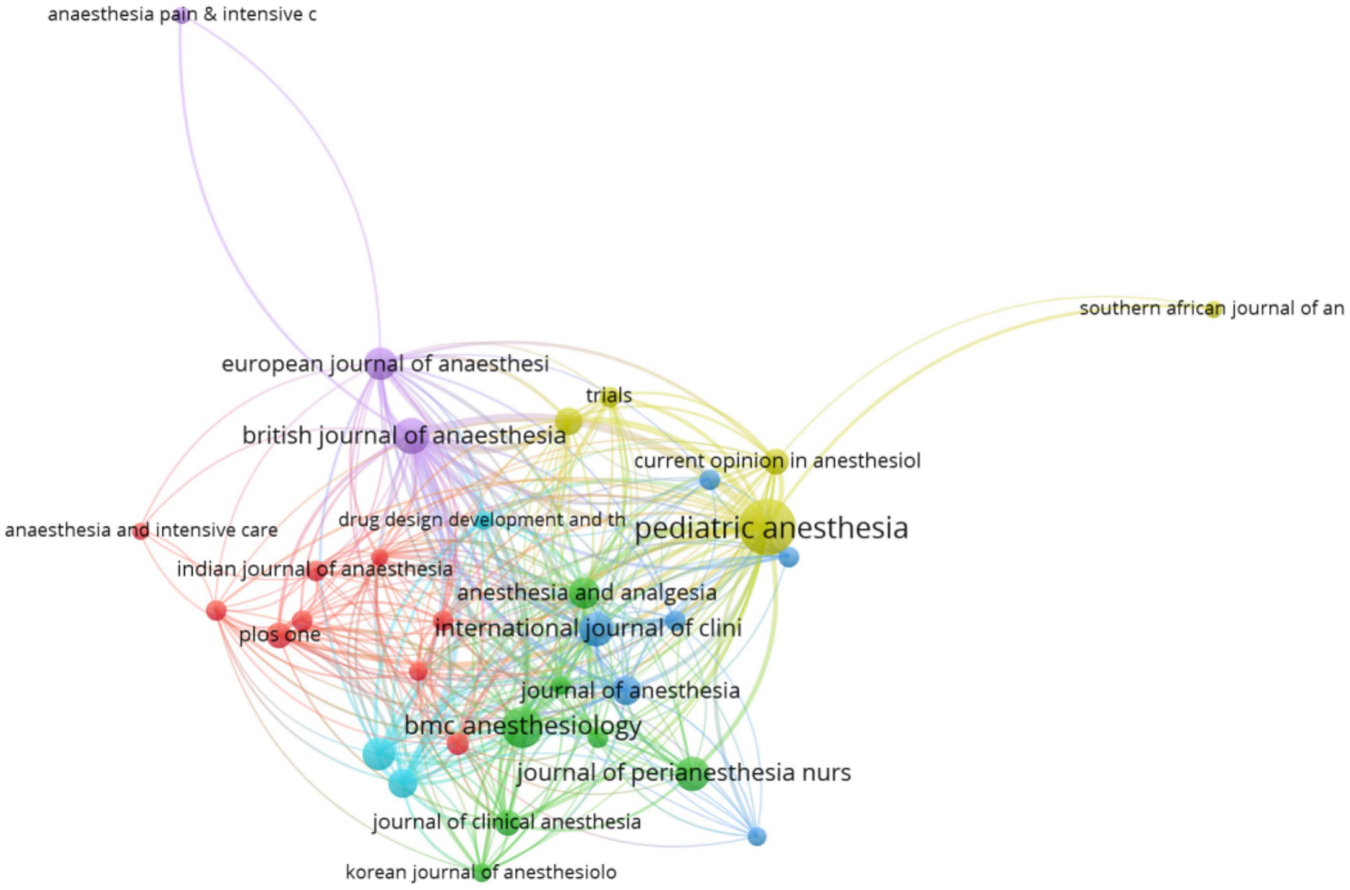
Network map of citation between sources in this field.

During that time, 409 journals published literature related to emergence delirium. The top 10 most productive journals in this area are shown in Table 3. These top 10 journals published a total of 214 documents, accounting for 23.46% of all. PEDIATRIC ANESTHESIA published the most document (*n* = 56). The second most popular journal was BMC ANESTHESIOLOGY, which has 30 publications. BRITISH JOURNAL OF ANAESTHESIA ranked No. 3 (n = 22) and was followed by INTERNATIONAL JOURNAL OF CLINICAL AND EXPERIMENTAL MEDICINE (*n* = 21), JOURNAL OF PERIANESTHESIA NURSING (*n* = 20). These journals focus on anesthesia and drugs. We use VOSviewer software to perform citation analysis of sources (Figure 10). Thirty-three sources were analyzed with a minimum of documents of over 5 times. BRITISH JOURNAL OF ANAESTHESIA had the most citations with 1,204 and was followed by PEDIATRIC ANESTHESIA (1,056 citations), MICROBIOLOGY AND MOLECULAR BIOLOGY REVIEWS(451 citations), NEUROPSYCHIATRIC DISEASE AND TREATMENT (402 citations), and ANESTHESIA AND ANALGESIA(400 citations). With regarded to source impact, the *h*-index was used to describe these journals’ importance. PEDIATRIC ANESTHESIA has the largest *h*-index of 21, followed by BRITISH JOURNAL OF ANAESTHESIA (*h*-index = 16), ANESTHESIA AND ANALGESIA (*h*-index =11), and BMC ANESTHESIOLOGY (*h*-index =10).

**Table 3:** Source impact of the top 10 journals publishing in this area.

Source	h_index	g_index	m_index	TC	NP
PEDIATRIC ANESTHESIA	21	29	1.909	1,056	55
BRITISH JOURNAL OF ANAESTHESIA	16	22	1.6	1,204	22
ANESTHESIA AND ANALGESIA	11	16	1	400	16
BMC ANESTHESIOLOGY	10	15	1.25	267	24
ACTA ANAESTHESIOLOGICA SCANDINAVICA	9	15	0.9	264	15
EUROPEAN JOURNAL OF ANAESTHESIOLOGY	9	14	0.818	216	18
CURRENT OPINION IN ANESTHESIOLOGY	8	12	0.727	270	12
JOURNAL OF CLINICAL ANESTHESIA	8	12	0.727	168	12
MEDICINE	8	11	1.143	147	17
MINERVA ANESTESIOLOGICA	8	12	0.727	159	13

## 4. Discussion

Bibliometrics is a branch of information science. It is a quantitative and qualitative analysis method that uses statistical data to describe or show the relationship between publications in a certain research field and objectively analyze the influence of research (Chen, 2017; Ninkov et al., 2022). However, other methods such as traditional retrospection, paired analysis, and experimental studies cannot achieve the same depth of analysis. Therefore, bibliometrics plays a very important role in improving literary ability and helping beginners to enter a certain field quickly.

Emergence delirium is a transient organic brain syndrome characterized by disturbances in attention, memory, feeling, thinking, psychomotor and sleep cycles. Clinically, emergence delirium usually presents as acute onset, mainly hyperactivity and agitation, and does not act according to instructions. Meanwhile, various degrees of involuntary movement occur. In serious cases, it may cause NSSI, fall from the bed and fall, and there are no clear diagnostic criteria. Waking agitation and emergence delirium are closely related and emergence delirium can be regarded as further development on the basis of waking agitation. The etiology and mechanism of emergence delirium remain unclear. Clinicians and researchers believe that the occurrence of emergence delirium is related to its risk factors. Possible risk factors currently considered include age, preoperative anxiety, pain stimulation, preoperative medication, mode of anesthesia, type of surgery, and time to recovery. Studies have shown that emergence delirium leads to an increased incidence of postoperative delirium (POD) (Franck et al., 2016). Early diagnosis of emergence delirium and early intervention can reduce the incidence of POD. Therefore, perioperative risk factors should be reduced as much as possible, and prevention should be emphasized to reduce the adverse prognosis caused by emergence delirium. At present, the main means of alleviating emergence delirium focus on prevention rather than treatment (Mason, 2017). With the popularization of anesthesia/surgery, it can be seen from the quantity distribution of literature that people pay more and more attention to emergence delirium in the past 10 years, and there are more and more research literature in the field of emergence delirium.

In this study, we performed a bibliometric analysis of trends and research hotspots in emergence delirium using data extracted from the Web of Science database using VOSviewer, CiteSpace and Bibliometrix software. Since the database was established, we have retrieved 912 original articles and reviews. As shown in Figure 2A, the number of annual publications shows an overall upward trend, especially after 2016, reaching a peak in 2021. The average annual growth rate of publications was 19.15%, Among all the retrieved articles, a total of 26,585 citations were received, with an average time cited per document of 14.29. Through the national citation map constructed by VOSviewer, it is found that the articles in the United States are the most cited countries, followed by China and then south Korea, and the correlation between them is the most concentrated place, indicating that they play an important role in the study of emergence delirium.

Table 1 shows that LEE JH is the author with the largest number of articles published in this field, with the highest H index and g index, with a total of 14 articles published in the past 10 years. The first article in the field was published in 2013, followed by another in the same year, demonstrating that LEE JH is one of the most productive and influential authors in the field. Among the authors who were co-cited and cited at least 20 times, the author with the highest intensity of connection with other authors was kain zn, whose paper was cited the most times, 448 times in total, indicating that his literature was of high quality and worthy of peer research.

Through the country/region distribution of literature, we can master the distribution of foreign research forces in this field, which provides a reference for our investigation. A total of 912 articles on emergence delirium were published in the last 10 years by research teams in 67 countries. The United States and China were the most prolific, each accounting for 22% of all countries’ publications, followed by South Korea (10%). There are 1,194 institutions involved in this area, Korea has the largest number of Yonsei Univ publications (n = 41), followed by Sichuan Univ (*n* = 31), Seoul Natl Univ (*n* = 27), Harvard Med Sch (*n* = 23), Korea Univ (*n* = 19). Although South Korea is lower than the US and China in terms of total national citation frequency, its two research institutions Yonsei Univ and Seoul Natl Univ occupy two of the top three places in the ranking of research institutions, which indicates that the average paper quality of these two institutions is slightly better than that of other national research institutions.

From the perspective of keyword analysis, co-occurrence analysis refers to the phenomenon that the same or different types of feature items appear at the same time. The purpose of co-occurrence analysis is to determine the popular fields and development direction of cooperative research (van Eck and Waltman, 2017). We constructed a co-occurrence knowledge map of keywords from Vosviewer to determine the keywords most cited in emergence delirium research (Figure 7), and the top 10 hot keywords are: “children” “emergence agitation” “delirium” “dexmedetomidine” “emergence delirium” “anesthesia” “propofol” “surgery” “sevoflurane” “general-anesthesia” (Table 4). It can be seen that children, emergence delirium, dexmedetomidine, anesthesia, surgery, propofol and sevoflurane are the hot and key research areas in recent 10 years. Emergence delirium is a common complication in pediatric surgery and anesthesia (Petre et al., 2021). Emergence delirium in children aged 2 to 12 years after general anesthesia is often reported in the literature, most of which have a short duration and are prone to contaminate the wound under the condition of unconsciousness, resulting in the removal of various drainage tubes and increasing the pressure of medical staff. The younger the age, the higher the incidence (Choi et al., 2011). In a prospective cohort study of children aged 3–10 years, younger age was associated with an increased risk of preoperative anxiety (Kain et al., 2000). At present, emergence delirium mainly focuses on preventive intervention strategies. Some scholars have proposed that preoperative anxiety will lead to the occurrence of postoperative negative behaviors. Preoperative anxiety relief is crucial for improving postoperative delirium (Banchs and Lerman, 2014), and the risk of preoperative anxiety is 3.5 times higher than that of emergence delirium (Dahmani et al., 2014). Drug therapy is of great significance for the prevention and treatment of emergence delirium in children. Dexmedetomidine is a highly selective α2 receptor agonist with sympathetic, analgesic, sedative and anti-anxiety effects (Gerlach and Dasta, 2007). Dexmedetomidine has been shown to reduce agitation from general anesthesia in children (Shukry et al., 2005; Patel et al., 2010) and is also frequently used in ICU patients with manic ventilator outage (Reade et al., 2009; Shehabi et al., 2010). Perioperative dexmedetomidine was associated with reduced intraoperative stress response, reduced postoperative opioid use, antiemetic therapy, and reduced pain (Gurbet et al., 2006; Tufanogullari et al., 2008; Ohtani et al., 2011; Blaudszun et al., 2012; Bekker et al., 2013). The use of dexmedetomidine reduced the amount of sevoflurane required during surgery (Di et al., 2017), and a meta-analysis also confirmed that dexmedetomidine reduced the incidence of emergence delirium in children under sevoflurane anesthesia (Zhu et al., 2015). LEE JH published the first paper during this period reporting that dexmedetomidine improved the quality of recovery after nasal surgery, resulting in more stable postoperative hemodynamics and smoother recovery of patients (Kim et al., 2013). For a long time, patients using inhalation general anesthesia have been more likely to develop agitation and emergence delirium(Urits et al., 2020). Studies have suggested that intravenous induction and maintenance anesthesia with propofol and remifentanil in children undergoing strabismic surgery have lower emergence delirium than sevoflurane induction and maintenance anesthesia (Chandler et al., 2013). A comparison between sevoflurane and intravenous anesthesia in patients undergoing dental surgery has also shown a higher incidence of post-anesthetic emergence delirium and postoperative pain (Kocaturk and Keles, 2018). Propofol is a common intravenous anesthetic. Continuous infusion of propofol throughout the anesthetic process reduced the incidence of emergence delirium more than a single infusion of propofol, and transitioning to propofol within 3 min after the end of sevoflurane anesthesia reduced the incidence of emergence delirium (van Hoff et al., 2015). Non-drug methods have become a new focus in the field of pediatric anesthesia to prevent emergence delirium. In pediatric patients, ophthalmology and otolaryngological surgery (including strabismus surgery and tonsillectomy) have been found to be associated with a high incidence of emergence delirium, especially otolaryngological surgery (Voepel-Lewis et al., 2003; Hino et al., 2017). In adult patients, emergence delirium rates were higher in patients undergoing spinal surgery, breast surgery, oral surgery, musculoskeletal surgery, otolaryngology surgery, and abdominal surgery (Lepousé et al., 2006; Radtke et al., 2010; Yu et al., 2010). Based on the mutant word monitoring function of Citespace, the results show that the keyword “halothane” has the strongest outbreak (intensity =8.51), citation outbreak from 2012 to 2016; The first 11 keywords mainly focused on the risk factors and evaluation of emergence delirium, and the last 7 keywords mainly focused on the control trial, dexmedetomidine prevention and treatment of emergence delirium.

**Table 4:** The top 10 most frequently used keywords.

Rank	Keywords	Occurrences
1	Children	255
2	Emergence agitation	249
3	Delirium	199
4	Dexmedetomidine	185
5	Emergence delirium	177
6	Anesthesia	173
7	Propofol	169
8	Surgery	154
9	Sevoflurane	153
10	General- anesthesia	141

By studying the distribution of source publications, it is helpful to find the main research positions in this field. According to the statistics, from 2012 to 2021, the literature was published in 409 different journals, and the top 10 journals were all influential in this field. Among them, the BRITISH JOURNAL OF ANAESTHESIA is the most cited journal (*n* = 1,204), PEDIATRIC ANESTHESIA journal has the largest number of publications and the highest H index. The number OF citations (*n* = 1,056) is second only to the BRITISH JOURNAL OF ANAESTHESIA, which is the most closely cooperated with other journals in this research field.

The study also had some limitations. First of all, this study only used the data in WoSCC database, and the literature time range was nearly 10 years. No statistical analysis was made on the literature published before 2012, which is bound to lead to the problem of incomplete analysis data. Secondly, due to the continuous update of data in WoSCC database, the results of bibliometric analysis often lag behind the actual research progress. In addition, only English articles are included in this study, which may reduce the number of articles retrieved. Finally, because our study is temporary, the number of relevant articles may also change over time.

## 5. Conclusion

This study used bibliometrics to collect 912 articles published between January 2012 and December 2021, revealing a collaborative network of contributing countries, institutions, journals and authors, and providing meaningful research results to illuminate trends and research hotspots in emergence delirium, and found that research in this area has been on the rise over the last decade. The United States dominates the field, with the largest number of publications (203 articles), the most cited publications and extensive international cooperation. In recent years, the research focus of emergence delirium has gradually shifted from the assessment of its risk factors (such as children, tonsil surgery, sevoflurane anesthesia) to the timely identification of high-risk patients, diagnosis and treatment with drug therapy and/or non-drug intervention, so as to achieve the purpose of prevention and control. Additionally, by processing a large number of scientific data and generating research influence to clarify the current research status, the study may attract the attention of more researchers and lay the foundation for subsequent mechanism and treatment research. We summarized the above aspects, and in the long run, it may have certain guidance for the existing and future treatment mode.

## Data availability statement

The original contributions presented in the study are included in the article/supplementary material, further inquiries can be directed to the corresponding authors.

## Author contributions

KW, JW, and RD contributed to the conception and design of the study. KW and JC extracted data sets from the Web of Science for statistical analysis and was a major contributor to writing the manuscript. JC participated in the interpretation of the research results. All authors contributed to the article and approved the submitted version.

## Funding

This work was supported by Medical Science and Technology Research Foundation of Guangdong Province, China (grant number B2022115).

## Conflict of interest

The authors declare that the research was conducted in the absence of any commercial or financial relationships that could be construed as a potential conflict of interest.

## Publisher’s note

All claims expressed in this article are solely those of the authors and do not necessarily represent those of their affiliated organizations, or those of the publisher, the editors and the reviewers. Any product that may be evaluated in this article, or claim that may be made by its manufacturer, is not guaranteed or endorsed by the publisher.
